# The Emerging Role of Fatty Acid Synthase in Hypoxia-Induced Pulmonary Hypertensive Mouse Energy Metabolism

**DOI:** 10.1155/2021/9990794

**Published:** 2021-08-17

**Authors:** Cuilan Hou, Juan Chen, Yuqi Zhao, Yanhua Niu, Shujia Lin, Shun Chen, Yanfang Zong, Xiaomin Sun, Lijian Xie, Tingting Xiao

**Affiliations:** Department of Cardiology, Shanghai Children's Hospital, Shanghai Jiao Tong University, No. 355 Luding Road, Shanghai 200062, China

## Abstract

**Aims:**

This study is aimed at examining whether fatty acid synthase (*FAS*) can regulate mitochondrial function in hypoxia-induced pulmonary arterial hypertension (PAH) and its related mechanism.

**Results:**

The expression of *FAS* significantly increased in the lung tissue of mice with hypoxia-induced PAH, and its pharmacological inhibition by C75 ameliorated right ventricle cardiac function as revealed by echocardiographic analysis. Based on transmission electron microscopy and Seahorse assays, the mitochondrial function of mice with hypoxia was abnormal but was partially reversed after C75 injection. *In vitro* studies also showed an increase in the expression of *FAS* in hypoxia-induced human pulmonary artery smooth muscle cells (HPASMCs), which could be attenuated by *FAS* shRNA as well as C75 treatment. Meanwhile, C75 treatment reversed hypoxia-induced oxidative stress and activated *PI3K/AKT* signaling. shRNA-mediated inhibition of *FAS* reduced its expression and oxidative stress levels and improved mitochondrial respiratory capacity and ATP levels of hypoxia-induced HPASMCs.

**Conclusions:**

Inhibition of *FAS* plays a crucial role in shielding mice from hypoxia-induced PAH, which was partially achieved through the activation of *PI3K/AKT* signaling, indicating that the inhibition of *FAS* may provide a potential future direction for reversing PAH in humans.

## 1. Introduction

Pulmonary arterial hypertension (PAH) is a disease that causes persistent increased mean pulmonary artery pressure (mPAH), vascular wall thickening, heart failure, and eventually death [1]. The pathogenesis of PAH involves vessel lumen narrowing and subsequent occlusion, intimal fibrosis, and the progressive development of concentric and plexiform lesions [2]. Previous studies have demonstrated that phosphodiesterase-2 [3], nitric oxide [4], and transforming growth factor-*β* (TGF-*β*) [5] were associated with the development of PAH. Despite an in-depth understanding of the pathogenesis and prognosis of PAH, there has still been no effective therapy demonstrated for patients with PAH.

Over the last 20 years, metabolic theory has been one of the leading theories explaining PAH. It points out that targeting fatty acid oxidation (FAO) by inhibiting malonyl CoA decarboxylase (MCD) or 3-ketoacyl CoA thiolase could relieve PAH phenotypes [6]. For example, human pulmonary arterial smooth muscle cells (HPASMCs) grow faster with PAH and rely not only on glycolysis but also on altered lipid metabolism to sustain their high proliferation rates [7]. A metabolic theory proposed by Paulin et al. states that the integration of upstream and downstream signals in the mitochondria is caused by PAH, which is similar to cancer [8]. This metabolic theory explains many features of PAH, including cell proliferation and apoptosis resistance [8]. Mitochondrial metabolism is deregulated in PAH, accompanied by pyruvate dehydrogenase inhibition and aerobic glycolysis [9]. Pulmonary arterial wall metabolic remodeling was also observed in PAH [6]. Singh et al. reported that fatty acid synthase (*FAS*) expression was increased in HPASMCs and the lung tissue of PAH rats. Moreover, they observed that the inhibition of *FAS* could relieve PAH phenotypes [10]. Despite these findings, further exploration of the PAH metabolic theory is still needed.

It has been reported that in hypoxic cardiomyocytes, siRNA-mediated inhibition of *FAS* could reduce hypertrophy, inflammation, apoptosis, and autophagy and improve glucose oxidation, mitochondrial membrane potential, and ATP levels [11]. *In vivo*, *FAS* inhibition by C75 (a *FAS* inhibitor) decreased the expression and activity of *FAS* and improved cardiac functions and mitochondrial membrane potential in monocrotaline-treated rats [11]. However, the effects of *FAS* on a hypoxia-induced PAH mouse model have not yet been reported, and its potential molecular mechanisms are still not well understood. Thus, the aim of this study was to investigate the effects of *FAS* on hypoxia-induced PAH mouse heart remodeling and mitochondrial function and to identify its underlying mechanism.

## 2. Materials and Methods

### 2.1. Chronic HyOA-Induced PAH Mice

Twenty-one C57BL/6 mice (eight weeks old) were purchased from the Shanghai Laboratory Animal Center (Shanghai, China) and grown under controlled conditions (45%–55% relative humidity, 22 ± 2°C, and 12 h dark-light cycles) with unrestricted access to food and water. The health and weight of these mice were continuously monitored throughout the experimental period.

**Figure 1:**
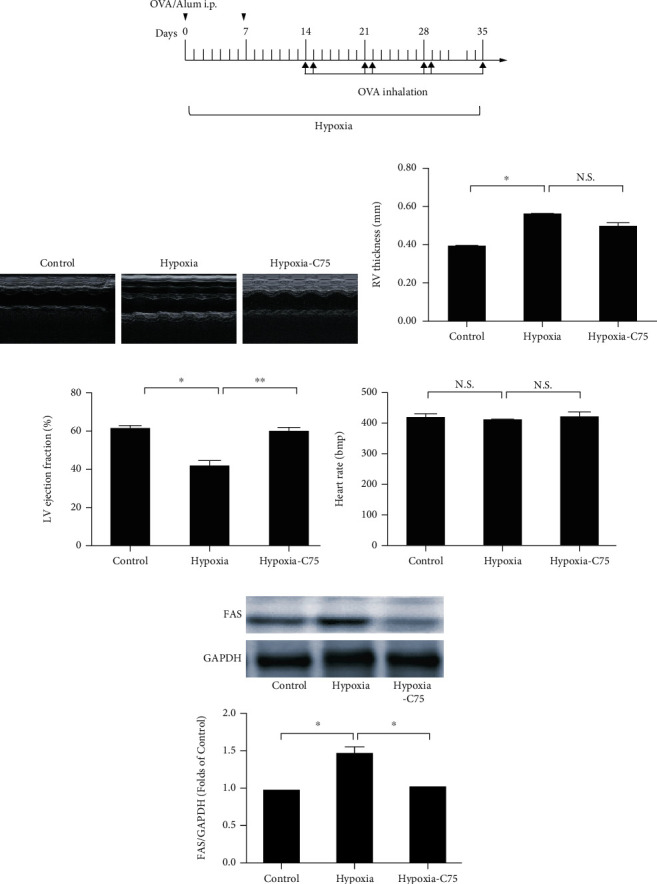
C75 protected against hypoxia-induced cardiac dysfunction. (a) Protocol for HySU-induced PAH in mice. (b–e) Representative of M-mode images and quantification of RV thickness, LV ejection fraction, and heart rate among the three groups (*n* = 6). (f) Representative Western blot images and quantification of the expression of *FAS* in mouse lung tissue (*n* = 4).

Fourteen mice received a single intraperitoneal injection of ovalbumin (OVA) (Sigma-Aldrich, USA) that was diluted to 1 mg/mL using sterile saline (Sigma-Aldrich, USA) with Imject Alum (Number: 77161, Thermo Fisher Scientific, USA; final dose, 50 *μ*g of OVA and 2 mg of Alum) on the first day of the first two weeks of treatment, as shown in Figure 1(a). Then, the animals were challenged with aerosolized OVA for 30 min at a concentration of 10 mg/mL twice per week on the first and last days of each week during the treatment period [12, 13]. Experimental animals were exposed to chronic hypoxia (10% O_2_) in a ventilated chamber for five weeks. The hypoxia-C75 groups received the *FAS* inhibitor C75 (200 *μ*g/kg/week, dissolved in 0.5% (dimethylsulfoxide) DMSO) for 5 weeks [14]. The hypoxia groups received an equivalent amount of 0.5% DMSO without C75 each week. The control mice (*n* = 7) received 0.5% DMSO and remained in a normoxic environment. At the end of the treatment period, samples were collected. The animal experiments were approved by the Shanghai Jiao Tong University Institutional Animal Care and Use Committee.

### 2.2. Electronic Microscopy Analysis

Transmission electron microscopy (TEM) for morphological analysis was performed at the Department of Physiology and Pathophysiology, School of Basic Medical Sciences, Fudan University, according to standard operating procedures. For morphological TEM, lung tissues were cut into 1 mm × 1 mm × 1 mm patches and fixed with ice-cold 2.5% glutaraldehyde at 4°C overnight. Ultrathin sections were stained with uranyl acetate and lead citrate. After sample preparation, 90–100 nm thick sections were mounted on 200 mesh copper grids and imprinted using an FEI Tecnai G2 Spirit TEM. In each sample, 6-8 visual fields were randomly selected, and then, the number of mitochondria was analyzed by ImageJ software. All analyses were performed blind to the observer.

### 2.3. Mitochondrial Function Analysis

Analysis of mitochondrial fuel usage was performed using Agilent Seahorse XF Cell Mito Stress Test Kits (Agilent Technologies, Santa Clara, CA, USA) according to the manufacturer's instructions. HPASMCs (2000/well) were seeded in Seahorse XFp96 cell culture miniplates 48 h before measurements and preincubated with C75 (25 mM for 24 h) and/or Ly294002 (10 *μ*M for 24 h). The day prior to the assay, a sensor cartridge was hydrated in Seahorse XF Calibrant at 37°C in a non-CO_2_ incubator overnight. On the day of the assay, XF Cell Mito Stress Test medium (XF Base Medium, 1 mM of pyruvate, 2 mM of glutamine, and 10 mM of glucose warmed up at 37°C and adjusted at pH 7.4 with 0.1 M of NaOH) was prepared. The abovementioned cells were transferred and incubated with the appropriate assay medium for 1 h in a non-CO_2_ incubator at 37°C. During the incubation time, the pouches containing the compounds (oligomycin, FCCP, trifluoromethoxy carbonyl cyanide phenylhydrazone, and rotenone/antimycin) were set at room temperature for 15 min. Compounds were resuspended with the prepared assay medium and then diluted in the same medium to obtain the following final concentrations: 10 *μ*M of oligomycin, 10 *μ*M of FCCP, and 5 *μ*M of rotenone/antimycin for the Mito Stress Test. The assay was conducted using a Seahorse XFp System and analyzed using Wave software (Agilent Technologies, Wilmington, USA).

### 2.4. Cell Culture and Cell Treatment

HPASMCs were purchased from ScienCell research laboratories (California, USA, Catalog #110) and cultured with smooth muscle cell medium (ScienCell research laboratories, Catalog #1101). For hypoxia assays, the HPASMCs were incubated under hypoxia conditions (3% O_2_, 5% CO_2_, and 92% N_2_) for 48 h. Hypoxic HPASMCs were treated with the pharmacological *FAS* inhibitor C75 (Sigma-Aldrich, USA) or *FAS* shRNA (60 nM) for 48 h.

### 2.5. Estimation of Fatty Acid Synthase Activity and Malonyl CoA and Palmitate Levels

Free fatty acids (FFAs) in the blood and HPASMC numbers were estimated via the protocol of a nonesterified FFA assay kit 96T (Nanjing Jiancheng Company, China). *FAS* activity was estimated according to a protocol previously described [15]. The level of malonyl CoA was estimated using a commercially available kit (CUSABIO, China), according to the manufacturer's protocol for *in vitro* studies [10].

### 2.6. Fluorescence Detection of ROS and Mitochondrial Superoxide

Intracellular reactive oxygen species (ROS) levels in HPASMCs were estimated using dihydroethidium (DHE) staining (Sigma-Aldrich, Germany). DHE powder was dissolved in dimethyl sulfoxide and diluted with phosphate buffer saline (PBS) at 55°C. HPASMCs were incubated with 1 *μ*M of DHE for 30 min and observed using a laser confocal microscope (Zeiss LSM710, Germany) at 488/610 nm. For the mitochondrial superoxide assay, 5 mM of MitoSOX reagent stock solution (Invitrogen, USA) was diluted in PBS to make a 5 *μ*M MitoSOX reagent working solution. HPASMCs were incubated with the MitoSOX reagent working solution for 30 min and observed under a laser confocal microscope using an excitation/emission wavelength of approximately 510/580 nm.

### 2.7. Real-Time PCR

Real-time PCR (RT-PCR) was used to detect the mRNA levels of Hif1, caspase 3, and caspase 9 in mice and *HIF1*, *CASPASE 3*, and *CASPASE 9* in HPASMCs. Briefly, RNA from mouse lung tissues and HPASMCs were extracted according to the manufacturer's protocol (Takara, Japan). The primer sequences are listed in Table 1.

### 2.8. Mitochondrial Extraction

Trypsinize and centrifuge HPASMCs for 5 minutes at 600 × g. Wash and resuspend the cells in ice-cold PBS; count and centrifuge the cells for 5 minutes at 600 × g at 4°C. Discard the supernatant. Add 1.5 mL of the prepared extraction buffer per 2-5 × 10^7^ cells. Incubate on ice for 10-15 minutes. Homogenize and transfer the supernatant liquid to a fresh tube. Centrifuge at 11,000 × g for 10 minutes at 4°C. Carefully remove the supernatant; for mitochondrial protein characterization, suspend the pellet with 100 *μ*L cell lysis reagent with protease inhibitor cocktail (1 : 100 (*v*/*v*)).

### 2.9. Immunofluorescence Staining

HPASMCs were seeded on confocal plates and pretreated with *FAS* (60 nM) lentivirus or its negative control, then fixed with 4% paraformaldehyde for 10 min, followed by permeabilization with 0.25% Triton X-100. Next, confocal plates were blocked with 5% bovine serum albumin for 60 min at room temperature and incubated with primary antibodies overnight at 4°C. Appropriate secondary antibodies were added and incubated for 1 h at room temperature. The images were captured by using a Zeiss LSM880 microscope and analyzed using ZEN software (Zeiss, Germany).

### 2.10. Cell Lysis and Western Blotting

To acquire protein for Western blotting analysis, cells were washed twice in PBS and incubated with cell lysate buffer at 4°C for 5 min with gentle rocking. The RIPA cell lysate buffer used contained protease inhibitors and phosphatase inhibitors. Proteins were resolved by SDS-PAGE and then transferred onto polyvinylidene fluoride membranes (Millipore, Bedford, MA, USA). These membranes were incubated with primary antibodies specific for Akt, p-Akt (Ser473), Bax, Bcl-2, Tom 20, and GAPDH (Cell Signaling Technology, USA), and VDAC1 and AIF (Abcam, Cambridge, MA, USA) at dilutions of 1 : 1000 in blocking buffer. Membranes were then incubated with secondary antibodies in blocking buffer at 1 : 2000 using horseradish peroxidase- (HRP-) conjugated secondary antibodies (Cell Signaling Technology, USA). After washing, the membranes were visualized using a chemiluminescent substrate (ECL). Densities of immunology bands were analyzed using a scanning densitometer (GS-800, Bio-Rad Laboratories, Hercules, CA, USA) coupled with Bio-Rad personal computer analysis software.

### 2.11. Statistical Analysis

Results are expressed as the mean ± SEM. Statistical analyses were performed using SPSS software, version 21.0 (SPSS, Inc., USA). Comparisons among groups were performed by one-way ANOVA. Paired data were evaluated by two-tailed Student's *t*-test. Statistical significance was defined as *P* < 0.05.

## 3. Results

### 3.1. Hypoxia-Induced PAH Mice Exhibit Cardiac Dysfunction

Echocardiogram studies were performed to check cardiac functions. Compared to control mice, hypoxia-treated mice showed increased right ventricular (RV) thickness and a reduction in LV ejection fraction (Figures 1(b)–1(d)), which was reversed in the C75 treatment group. The average heart rate among all three groups was about 416 bpm, and there were no significant differences between these groups (Figure 1(e)). Chronic hypoxia treatment can induce pulmonary artery remodeling; the mouse vascular medial thickness to total vessel size was much larger than the control mice (Fig. s1). Compared to control mice, the expression of *FAS* (one of the most important enzymes of fatty acid synthesis) increased in the hypoxia groups, which was partially reversed after C75 treatment (Figure 1(f)).

### 3.2. Long Hypoxia Exposure Induces Lung Mitochondrial Dysfunction

**Figure 2:**
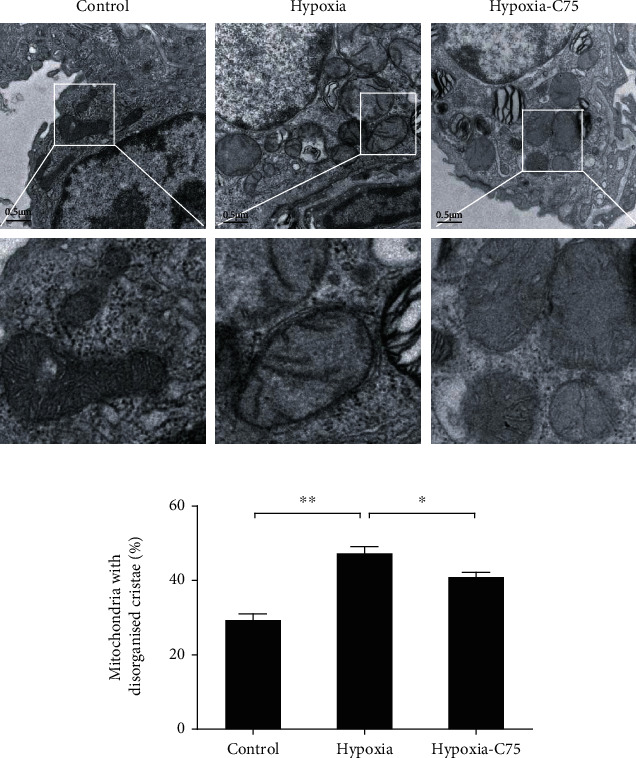
C75 protected against hypoxia-induced lung mitochondrial dysfunction. The ultrastructure of the mice was examined by TEM. (a) Representative of TEM images. Scale bar = 0.5 *μ*m. (b) Quantitative analysis of the mitochondria with disorganized cristae among the three groups (*n* = 3).

Next, mitochondrial ultrastructure was detected using a TEM. The lungs of mice with long-term exposure to hypoxia exhibited ultrastructural changes in their mitochondria, including irregular arrangement, vacuolation, and loss of cristae (Figure 2(a)). Compared to control mice, the percentage of hypoxia-treated mice with mitochondria having disorganized cristae was significantly higher, which was partially reversed in the C75 treatment group (Figure 2(b)).

### 3.3. C75 Incubation Activated PI3K/Akt Signaling and Apoptosis

**Figure 3:**
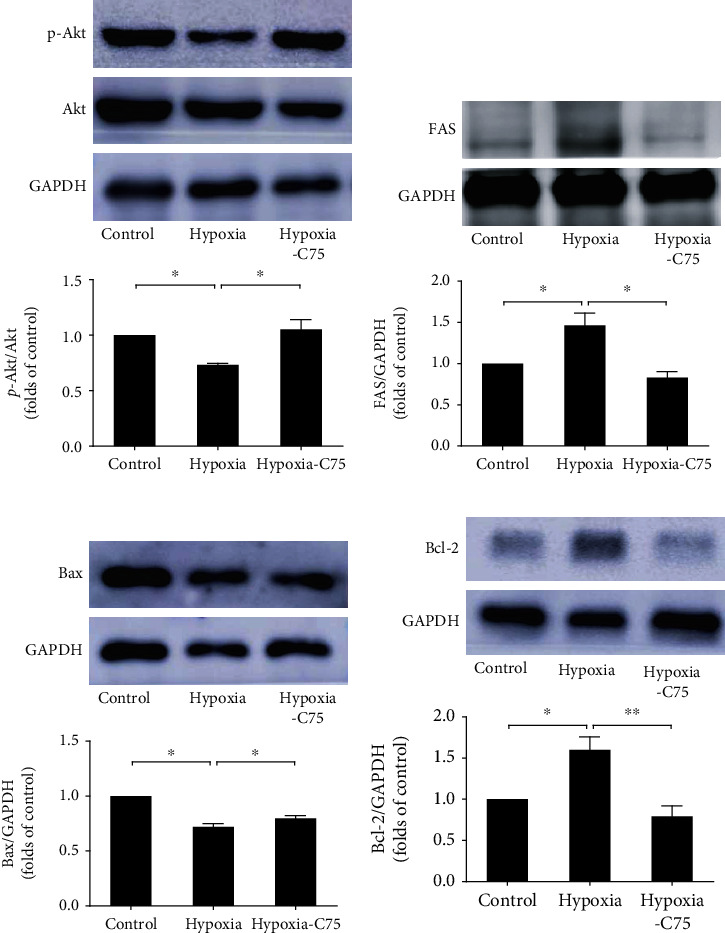
C75 protected against hypoxia-induced apoptosis through PI3K/Akt signaling. (a–d) Representative Western blot images and quantification of the expression of p-Akt, *FAS*, Bax, and Bcl-2 in the mouse tissues (*n* = 4).

Next, HPASMCs were used *in vitro* to detect the effects of hypoxia on survival and apoptosis-related proteins (PI3K/Akt signaling, *FAS*, Bax, and Bcl-2). *In vivo* experiments showed that compared to the control group, p-Akt (Ser473) and Bax signaling were decreased in the hypoxia groups, which were reversed after C75 treatment (Figures 3(a) and 3(c)). *FAS* and Bcl-2 (one of the key antiapoptosis proteins) increased in these hypoxia groups, and this was reversed after C75 treatment (Figures 3(b) and 3(d)).

### 3.4. C75 Incubation Activates Mitochondria-Dependent Apoptosis

**Figure 4:**
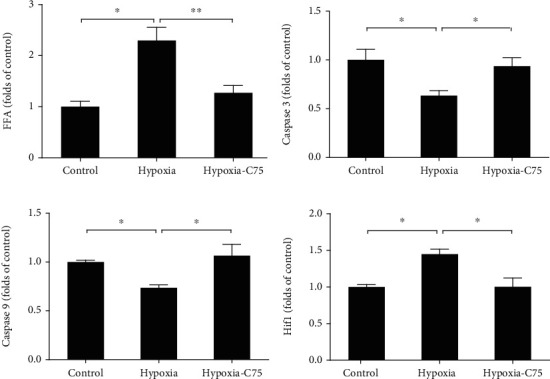
C75 played a protective role in mitochondria-independent apoptosis. (a) Quantification of FFA (palmitate) levels of mouse serum samples (*n* = 4). (b–d) Quantification of mRNA levels of caspase 3, caspase 9, and Hif1 in the hypoxia-induced mice (*n* = 5).

**Figure 5:**
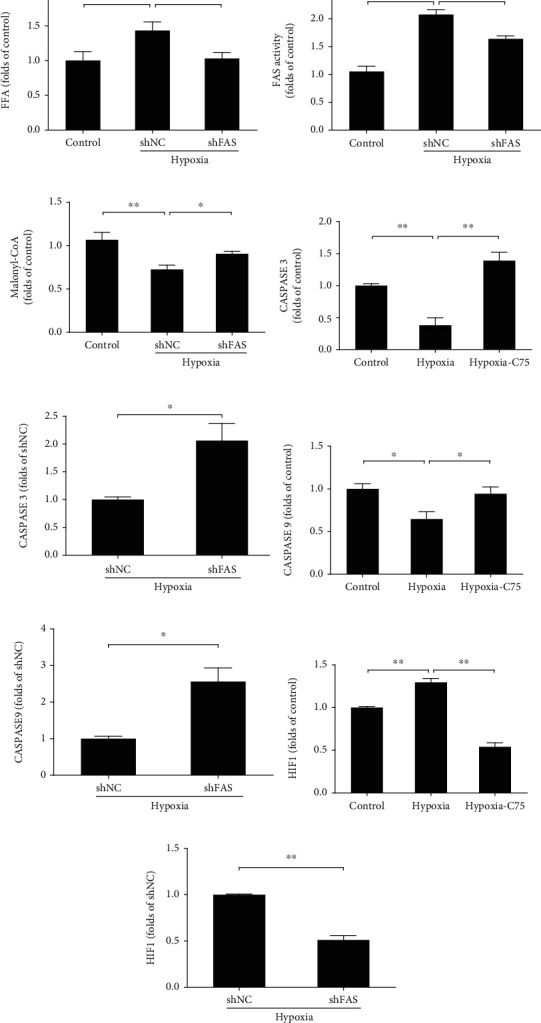
Effect of C75 and *FAS* shRNA treatment on HPASMCs. (a) Quantification of FFA (palmitate) levels in HPASMCs (*n* = 8). (b, c) Quantification of *FAS* activity and malonyl CoA levels in the HPASMCs (*n* = 7). (d–i) Quantification of mRNA levels of *CASPASE 3*, *CASPASE 9*, and *HIF1* upon C75 or shFAS treatment in the HPASMCs (*n* = 4).

Compared with the control group, the FFA levels (palmitate) were increased significantly after five weeks of hypoxia treatment, which were partially reversed upon C75 treatment (Figure 4(a)), and the same tendency was observed in hypoxic HPASMCs (Figure 5(a)). A concentration of 60 nM of shRNA decreased the enhanced *FAS* activity of hypoxic HPASMCs (Figure 5(b)). This increased *FAS* activity was also supported by decreased malonyl-CoA levels and increased FFA levels (palmitate level) in hypoxic HPASMCs which were significantly attenuated by *FAS* inhibition (Figures 5(a) and 5(c)).

Next, we found that the caspase family plays a crucial role in mitochondria-dependent apoptosis of PAH. We found that the mRNA levels of caspase 3 and caspase 9 were both decreased in five-week hypoxia-treated mice, compared with the relevant controls, which were partially reversed upon C75 treatment (Figures 4(b) and 4(c)). In terms of Hif1 that plays key roles in the regulation of hypoxia condition, we observed that C75 treatment could also decrease Hif1 mRNA levels (Figure 4(d)).

**Figure 6:**
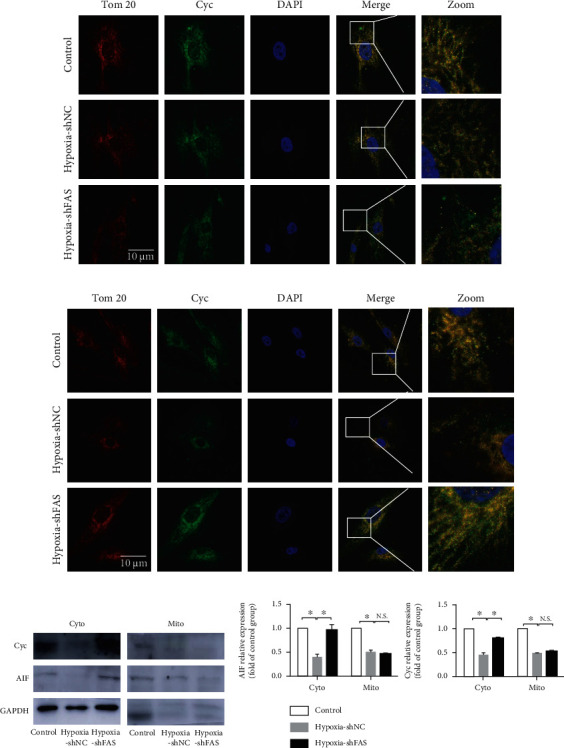
*FAS* activated mitochondria-dependent apoptosis in HPASMCs. (a, b) Representative images of Cyc and AIF colocalization with Tom 20 in HPASMCs (*n* = 6). Scale bar = 10 *μ*m. (c, d) Cyc and AIF released from the mitochondria to the cytoplasm were analyzed by Western blotting. VDAC1 was used as a loading control for mitochondria gradient. GAPDH for cytosolic gradient (*n* = 3). Values are the mean ± SE. *P* < 0.05 was considered significant.

We also used HPASMCs *in vitro* to test the protective role of C75 in mitochondria-dependent apoptosis. We found that both *CASPASE 3* and *CASPASE 9* mRNA levels were decreased upon 48 h hypoxia induction and reversed after C75 treatment of shFAS infection (Figures 5(d)–5(g)). Both C75 treatment and 60 nM shFAS infection could decrease *HIF1* mRNA levels in hypoxic HPASMCs (Figures 5(h) and 5(i)). Meanwhile, we found that apoptosis inducing factor (AIF) and cytochrome C (Cyc) were released from mitochondria to cytoplasm after 60 nM shFAS infection via immunofluorescent staining (Figures 6(a) and 6(b)) and Western blotting (Figures 6(c) and 6(d)). 60 nM shFAS infection increased the expression levels of Cyc and AIF in cytoplasm (cyto) after chronic hypoxia treatment. The Cyc and AIF levels were decreased significantly after hypoxia pretreatment in mitochondria (mito), but there was no significance between the 60 nM shFAS infection group and its negative control (Figures 6(c) and 6(d)).

### 3.5. C75 Reversed Hypoxia-Induced Oxidative Stress

**Figure 7:**
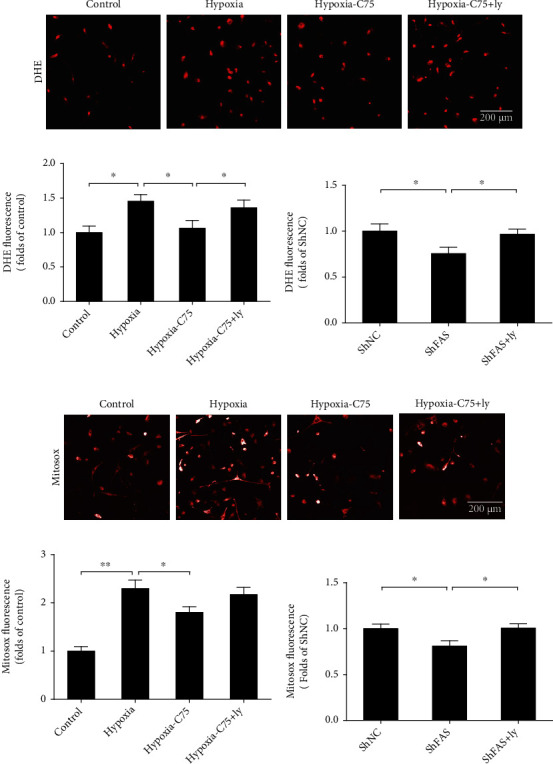
C75 protected against hypoxia-induced oxidative stress. (a, b) Representative DHE images and quantitative analysis of DHE intensity after incubation with C75 and Ly294002 in HPASMCs (*n* = 8). (c) Quantitative analysis of DHE intensity after transfection with *FAS* knockdown virus in HPASMCs (*n* = 6). (d, e) Representative MitoSOX images and quantitative analysis of MitoSOX intensity after incubation with C75 and Ly294002 in HPASMCs (*n* = 6). (f) Quantitative analysis of MitoSOX intensity after transfection with *FAS* knockdown virus in HPASMCs (*n* = 6). C75: 25 mM for 24 h; Ly294002: 10 *μ*M for 24 h. Values are the mean ± SE. *P* < 0.05 was considered significant.

Mitochondrial dysfunction often results in an increase of ROS. To quantify the level of ROS, a DHE fluorescence assay was used. DHE intensity was elevated in the hypoxia groups relative to that of the control, which was reversed after C75 treatment. After incubation with the *PI3K/AKT* inhibitor Ly294002, the antioxidative effect of C75 was eliminated (Figures 7(a) and 7(b)). Additionally, *FAS* was knocked down via lentivirus, resulting in a decrease of DHE intensity in hypoxia groups. Similarly, after incubation with Ly294002, the antioxidative effect of C75 was eliminated (Figure 7(c)). These results indicated that C75 could protect against hypoxia-induced oxidative stress partially through *PI3K/AKT* signaling.

Meanwhile, MitoSOX intensity was elevated in the hypoxia group relative to that of the control, which was reversed after C75 treatment. After incubation with Ly294002, the antioxidative effect of C75 was eliminated (Figures 7(d) and 7(e)). Additionally, when *FAS* was knocked down via lentivirus, this resulted in the decrease of MitoSOX intensity in the hypoxia group. After incubation with Ly294002, antioxidative effects of C75 were eliminated (Figure 7(f)). These data demonstrated that hypoxia may induce oxidative burden and mitochondrial superoxide production that are correlated with mitochondrial dysfunction, and C75 incubation was able to partially prevent against hypoxia-induced oxidative stress.

### 3.6. C75 Incubation Reverses Hypoxia-Induced Mitochondrial Respiratory Disability

**Figure 8:**
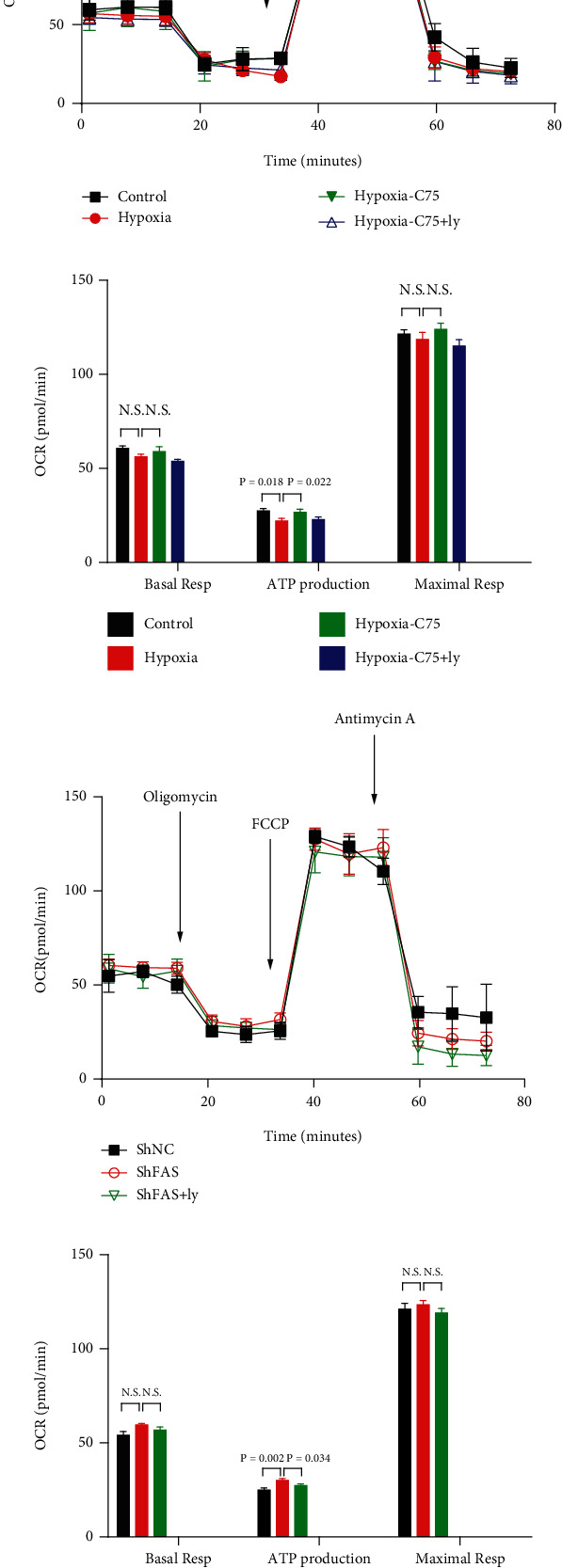
C75 protected against hypoxia-induced mitochondrial respiration dysfunction. (a, b) Measure of OCR and respective quantitative analysis of HPASMCs after incubation with C75 and Ly294002 (*n* = 8). (c, d) Measure of OCR and respective quantitative analysis of HPASMCs after transfection with *FAS* knockdown virus (*n* = 6). C75: 25 mM for 24 h; Ly294002: 10 *μ*M for 24 h; FCCP: trifluoromethoxy carbonyl cyanide phenylhydrazone. Values are the mean ± SE. *P* < 0.05 was considered significant.

The effects of hypoxia on mitochondrial respiratory capacity were then determined via the Seahorse assay. Compared to the control group, the HPASMCs of the hypoxia groups exhibited decreased ATP production, which was reversed by C75 treatment. The basal respiration and maximal respiration trends were not significantly different among the three groups. After incubation with Ly294002, the ability of C75 to enhance ATP production remained (Figures 8(a) and 8(b)). Additionally, when *FAS* was knocked down via lentivirus, it resulted in the increase of ATP production in the hypoxia group, which was reversed after incubation with Ly294002. The basal respiration and maximal respiration trends were not significantly different between these three groups (Figures 8(c) and 8(d)).

## 4. Discussion

There is increasing evidence that metabolic dysfunction underlies the pathogenesis of PAH [6, 10, 11], particularly altered lipid metabolism. However, there have been no studies on *FAS* inhibition in hypoxia-induced PAH mice. This study demonstrated that pharmacological inhibition by C75 can ameliorate right ventricle cardiac function in hypoxia-induced PAH mice, as revealed by echocardiographic analysis. TEM imaging and Seahorse assays revealed that mitochondrial function was disturbed in the hypoxia group, which was then partially reversed by C75 incubation. *In vitro*, C75 incubation reversed hypoxia-induced oxidative stress, activated PI3K/Akt signaling, and regulated mitochondria-dependent apoptosis. In all, the inhibition of *FAS* had a crucial role in shielding hypoxia-induced PAH. This was partially accomplished through the activation of PI3K/Akt signaling, indicating that the inhibition of *FAS* may serve as a potential means for reversing PAH.

It has been reported that inhibiting FAO was beneficial for right ventricle hypertrophy. FAO uses 12% more oxygen than glucose oxidation to produce the same amount of ATP [16]. Carnitine palmitoyltransferase-1 (CPT-1) is responsible for the transport of fatty acids into the mitochondria for *β* oxidation [17]. Singh et al. showed that there was a significant increase in CPT-1, indicating increased FAO in hypoxia cardiomyocytes, which was attenuated by *FAS* inhibition [11]. This reduced FAO could be the reason behind the protective effect of *FAS* inhibition on cardiac hypertrophy [11]. In our study, we also found that *FAS* significantly increased in the lung tissue of hypoxia-treated PAH mice and that its pharmacological inhibition using C75 ameliorated the right ventricle cardiac function (Figures 1(b)–1(f)). These data indicated that the *FAS* inhibitor C75 improved cardiac function in both MCT-induced PAH rats and hypoxia-induced PAH mice.

The mitochondria are the main metabolic sensors of a cell, which determine both ATP production and apoptosis [18]. A disturbance in mitochondrial ROS generation and energy production is a characteristic of PAH [19]. Singh et al. pointed out that mitochondrial function improved markedly with increased ATP levels following *FAS* inhibition in PAH rats [10]. Another feature of mitochondrial impairment is the hyperpolarization of the mitochondrial membrane. Singh et al. also reported that *FAS* inhibition restored the mitochondrial membrane potential and increased the translocation of apoptosis inducing factor antibody and Cyc from mitochondria, indicating the induction of apoptosis in PAH rats [10]. We found that the mitochondrial function was disturbed in hypoxia-induced PAH mice. The percentage of mitochondria with disorganized cristae ultrastuctures significantly increased in the hypoxia group, which was partially reversed after C75 injection (Figures 2(a) and 2(b)). Our data on PAH mice agreed with previous studies, wherein mitochondrial dysfunction was demonstrated by the depolarization of the mitochondrial membrane and impaired ATP generation in cardiac dysfunction [20, 21]. Meanwhile, we also noticed that oxidative stress (including mitochondrial oxidative stress) products were activated after long-term hypoxia, which was reversed by C75 incubation (Figures 7(a)–7(c)). MitoSOX fluorescence intensity in hypoxic HPASMCs was increased, which was decreased after C75 incubation or shFAS infection (Figures 7(d) and 7(e)). This is in line with the latest reports; Sheak et al. reported PKC*β* and mitochondrial reactive oxygen species signaling to increase pulmonary vasoconstrictor reactivity in chronically hypoxic neonates [22]. Hang et al. reported that hypoxia increased BPA levels of MitoSOX-detected superoxide and caused changes in NOX2 and SOD2 expression similar to COMP siRNA, and exogenous COMP (0.5 *μ*M) prevented the effects of hypoxia [23]. This indicated that C75 may protect against PAH through antioxidative stress and enhancement of mitochondrial function. These results were also supported by the results of the *in vivo* studies in which there was an increased expression of the antiapoptotic gene Bcl-2 and a decreased expression of the proapoptotic gene Bax in hypoxia-induced PAH mice. Both effects were significantly reversed by *FAS* inhibition (Figures 3(c) and 3(d)).

Mitochondria-dependent apoptosis is a potential target for PAH [24]. The Bcl-2 family plays a crucial role in the regulation of mitochondria-dependent apoptosis and interacts with pro- and antiapoptotic members (such as Bax) in deciding cell fate [25]. Singh et al. found an increased presence of cytosolic Cyc and caspase 3 activity in the right ventricle of MCT-treated rats and hypoxic cardiomyocytes, respectively, which was reversed by *FAS* inhibition [11]. We also found that caspase 3 and caspase 9 were decreased upon hypoxia induction both in vitro and in vivo, which were partially reversed by C75 treatment or shFAS infections. The expression levels of Cyc and AIF in cytoplasm (cyto) were increased after 60 nM shFAS infection and decreased significantly after hypoxia pretreatment in mitochondria (mito) (Figures 4–6). Based on the above literature review and our results, C75 may play an important role in mitochondria-dependent apoptosis of PAH. The protective capacity of C75 in PAH needs further study.

Activation of PI3K/Akt has been shown to play a major role in cardiomyocyte survival and function, as well as in the prevention of apoptosis [26–28]. Singh et al. reported that there was a significant decrease in p-PI3K and p-Akt expression in hypoxia cardiomyocytes, which was significantly reversed by *FAS* inhibition, and a significant increase in the expression and activity of *FAS* in hypoxia primary cardiomyocytes, which was attenuated by C75 treatment [10]. In the present study, we also found that p-Akt activation was partially blocked in our hypoxia-induced HPASMC model (Figure 3(a)). Interestingly, the protective effects of C75 on oxidative stress were weakened after incubation with Ly294002 (Figure 7). Moreover, the ability of C75 to protect mitochondrial respiratory ability was also weakened (Figure 8). All of the above results suggested that the inhibition of *FAS* had crucial roles in shielding cells from hypoxia-induced PAH, and this occurs partially through the activation of PI3K/Akt signaling.

## 5. Conclusions

In conclusion, the present study revealed that there was a significantly increased expression of *FAS* in hypoxia-induced mouse lung tissue, and its pharmacological inhibition using C75 ameliorated right ventricle cardiac and mitochondrial functions. *In vitro*, there was also a significant increase in the expression of *FAS* in hypoxia HPASMCs, which was attenuated by *FAS* shRNA as well as C75 treatment. Meanwhile, C75 reversed hypoxia-induced oxidative stress, activated PI3K/Akt signaling, and regulated mitochondria-dependent apoptosis. All of these data indicate that the inhibition of *FAS* played a key role in shielding hypoxia-induced PAH, and it occurs partially through the activation of PI3K/Akt signaling. The inhibition of *FAS* may provide a potential future direction for reversing PAH.

### 5.1. Shortcoming

It is better to confirm the protective effects of C75 on right ventricular pressure (RVP), which is caused by in hypoxia-induced PAH mice. It is difficult to measure the pressure of the right ventricle in mice. By determining the thickness of the tube wall and right ventricular remodeling (Fig. s1), we suggested that the model of PAH was successfully constructed. The lack of direct evidence of the protective effects of *FAS* inhibitors on RVP is the biggest defect of this work. Singh et al. reported that in a monocrotaline-induced PAH model, there was an increase in RVP of PAH rats (about 55 mmHg), which was significantly reversed by *FAS* inhibition (C75 treatment groups were about 40 mmHg) [10]. Singh et al. also showed that cardiac hypertrophy markers were decreased following *FAS* inhibition in MCT-induced pulmonary arterial hypertension [10]. It is better to measure RVP levels among the three groups: control, hypoxia, and hypoxia-C75. We will further study the therapeutic effect of *FAS* and the protective effects of C75 on RVP caused by in hypoxia-induced PAH.

## Figures and Tables

**Table 1:** The primers used in construction of the plasmid, point mutation, and qPCR.

HIF1-H-R	GCAGCAACGACACAGAAACT
HIF1-H-F	TGCAGGGTCAGCACTACTTC
CASPASE 3-H-F	ACTGGACTGTGGCATTGAGA
CASPASE 3-H-R	ATAACCAGGTGCTGTGGAGT
CASPASE 9-H-F	GCTTAGGGTCGCTAATGCTG
CASPASE 9-H-R	TGCAAGATAAGGCAGGGTGA
Caspase 9-M-F	GTGAAGAACGACCTGACTGC
Caspase 9-M-R	TCTCAATGGACACGGAGCAT
Hif1-M-F	TGAGCTTGCTCATCAGTTGC
Hif1-M-R	GCTCCGCTGTGTGTTTAGTT
Caspase 3-M-F	GGGCCTGAAATACCAAGTCA
Caspase 3-M-R	TCCCATAAATGACCCCTTCA

## Data Availability

The data used to support the findings in this study are available from the corresponding authors upon request.

## Supplementary Materials

Supplementary Materials Fig. s1: HE staining of the mouse lung tissues. Representative images of HE staining in the mouse lung tissues (*n* = 3). Scale bar = 10 *μ*m.
