# Supplementary figures and images for: The Emerging Role of Fatty Acid Synthase in Hypoxia-Induced Pulmonary Hypertensive Mouse Energy Metabolism
Source: Oxid Med Cell Longev. 2021 Aug 17;2021:9990794. doi: 10.1155/2021/9990794 (PMC8387195; doi:10.1155/2021/9990794)

**Control**

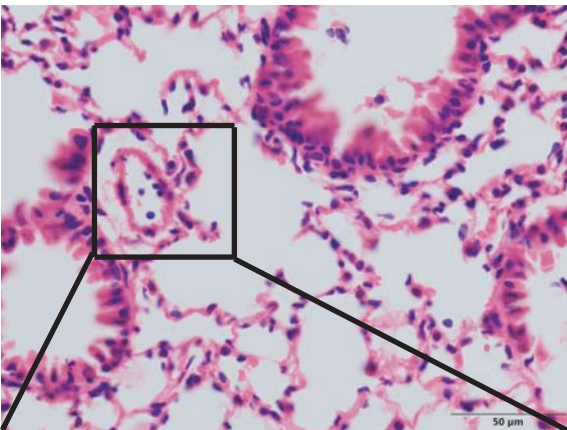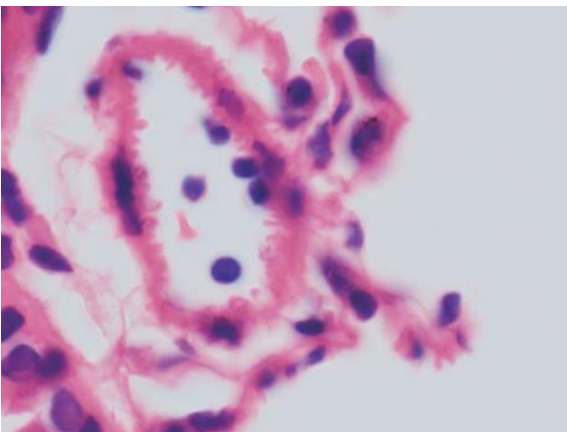

**Hypoxia**

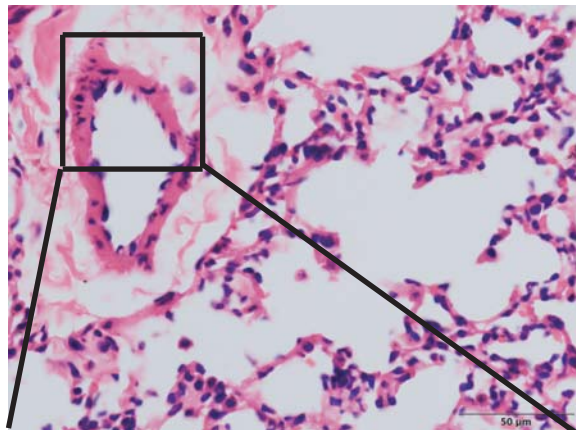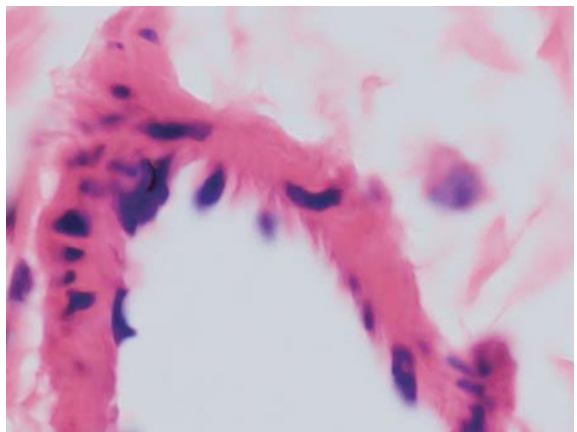

Supplement: Supplementary Materials — Fig. s1: HE staining of the mouse lung tissues. Representative images of HE staining in the mouse lung tissues (n = 3). Scale bar = 10 μm. [file 9990794.f1.pdf]
